# Editorial for Nanoparticle-Based (Bio)Sensors for Biomedical and Environmental Monitoring

**DOI:** 10.3390/mi17060715

**Published:** 2026-06-11

**Authors:** Stella Girousi

**Affiliations:** Laboratory of Analytical Chemistry, School of Chemistry, Aristotle University of Thessaloniki, 54124 Thessaloniki, Greece; girousi@chem.auth.gr

Nanoparticle-based biosensors are redefining the future of biomedical diagnostics and environmental monitoring. As the world faces growing health crises, emerging infectious diseases, and increasing environmental pollution, the need for rapid, sensitive, and portable detection systems has never been greater. Conventional analytical methods, although reliable, are often expensive, time-consuming, and dependent on sophisticated laboratory infrastructure. In this context, nanoparticle-enabled biosensors emerge as a transformative technology capable of delivering accurate results in real time while remaining cost-effective and user-friendly [1].

Nanoparticles possess unique physicochemical properties such as a high surface-to-volume ratio, enhanced electrical conductivity, optical tunability, and catalytic activity. These features significantly improve the sensitivity and selectivity of biosensors. Gold nanoparticles, quantum dots, magnetic nanoparticles, and carbon-based nanomaterials are among the most widely utilized materials in sensor fabrication. Their integration with biological recognition elements—including enzymes, antibodies, DNA, and aptamers—has enabled the development of advanced biosensing platforms capable of detecting diseases, toxins, pathogens, and environmental contaminants at extremely low concentrations [2].

In the biomedical field, nanoparticle-based biosensors have revolutionized disease diagnostics. Early detection of illnesses such as cancer, diabetes, cardiovascular disorders, and viral infections is critical for effective treatment and patient survival. Nanobiosensors offer rapid and minimally invasive diagnostic solutions that can operate even at the point of care. During global health emergencies such as the COVID-19 pandemic, nanoparticle-assisted diagnostic kits demonstrated the importance of portable and highly sensitive detection technologies [3]. These innovations not only reduced diagnostic delays but also improved healthcare accessibility in resource-limited regions.

Equally important is the role of nanoparticle-based biosensors in environmental monitoring. Industrialization, urbanization, and agricultural activities have introduced harmful pollutants into air, water, and soil ecosystems. Detecting heavy metals, pesticides, toxic gases, and microbial contaminants requires technologies capable of continuous and on-site monitoring. Nanoparticle-enabled sensors provide exceptional responsiveness and precision, allowing authorities and researchers to identify pollutants before they cause irreversible ecological damage. Their portability and low power requirements make them especially valuable for remote and underserved areas where conventional laboratory testing is impractical [4].

Despite these promising advancements, several challenges remain. Issues concerning nanoparticle toxicity, long-term environmental impact, reproducibility, and large-scale commercialization must be carefully addressed. Regulatory frameworks and standardized testing protocols are also essential to ensure the safety and reliability of these technologies. Furthermore, interdisciplinary collaboration among material scientists, biologists, engineers, and policymakers is necessary to bridge the gap between laboratory innovation and real-world application.

The future of nanoparticle-based biosensors is undeniably promising. As nanotechnology continues to evolve, these sensors are expected to become smarter, more affordable, and increasingly integrated with artificial intelligence and wireless communication systems. Such advancements could enable real-time health tracking, personalized medicine, and intelligent environmental surveillance networks. Ultimately, nanoparticle-based biosensors represent not only a scientific breakthrough but also a powerful tool for building a healthier and more sustainable world [5].

Recent years have witnessed remarkable progress in the development of nanostructured materials, electrochemical sensing platforms, molecular monitoring systems, and simulation-driven device engineering. These advances continue to reshape the landscape of analytical science by enabling highly sensitive, selective, rapid, and miniaturized sensing technologies for applications ranging from environmental monitoring to biomedical diagnostics and physiological assessment.

The collection of contributions presented in this issue of *Micromachines* highlights the interdisciplinary nature of contemporary sensor research. The published works collectively demonstrate how innovations in nanomaterials, electrode engineering, computational modeling, and molecular systems integration are driving the next generation of analytical devices and monitoring technologies.

Environmental sensing and pollutant monitoring remain central themes in this collection. Keramari et al. [Contribution 1] present the development and application of a nanostructured Mn_3_O_4_-based sensor for the determination of heavy metals in water and wastewater. Their work demonstrates the growing importance of transition-metal oxide nanostructures in achieving enhanced electrochemical sensitivity and stability for environmental analysis. The proposed sensor platform contributes to ongoing efforts toward affordable, portable, and efficient water quality monitoring systems capable of addressing critical environmental and public health challenges.

Similarly addressing environmental remediation and electrochemical performance, Kaludjerović et al. [Contribution 2] investigate a holmium metal nanoparticle PbO2 anode formed by electrodeposition for the efficient removal of the insecticide acetamiprid, while simultaneously improving the oxygen evolution reaction. This study highlights the dual functionality of advanced electrode materials in pollutant degradation and electrochemical process optimization. The incorporation of rare-earth metal nanoparticles into electrode architectures illustrates how material engineering can significantly improve catalytic activity and operational efficiency.

Nanostructured gold materials continue to attract substantial interest in biosensing due to their unique electrical conductivity, biocompatibility, and surface properties. In this issue, Lingden et al. [Contribution 3] report on a nanoporous gold nanoparticle-modified electrode for the detection of endotoxins. Their work demonstrates how porous nanostructured metallic interfaces can enhance analyte interaction and signal transduction, enabling sensitive detection platforms with potential applications in biomedical diagnostics, pharmaceutical quality control, and healthcare monitoring.

The growing integration of computational modeling and nanoscale electronics is represented by the contribution of Tamersit et al. [Contribution 4], who perform a quantum simulation study of ultrascaled label-free DNA sensors based on sub-10 nm dielectric-modulated TMD FETs. Their work explores sensitivity enhancement through device downscaling and provides valuable theoretical insights into the operation of next-generation nanoscale biosensors. Such simulation-driven approaches are increasingly important for accelerating device optimization and reducing experimental development costs while advancing the miniaturization of sensing technologies.

Optical sensing methodologies are also prominently featured in this collection. Epuran et al. [Contribution 5] investigate UV–Vis detection of thioacetamide through comparative evaluation of a Mn(III)-porphyrin, gold colloid, and their complex. By balancing the performance characteristics of these sensing materials, the authors identify strategies for optimizing sensitivity and analytical response. Their work contributes to the broader field of optical and plasmonic sensing, where hybrid material systems are being designed to improve selectivity and detection efficiency for chemical monitoring applications.

The issue additionally includes a comprehensive review by Shellaiah [Contribution 6] on biomedical, biomolecular, and environmental monitoring applications of cysteamine functionalized nanomaterials. This review provides a broad overview of functionalization strategies and demonstrates the versatility of cysteamine-based nanostructures in sensing applications. By summarizing recent developments across multiple analytical domains, the review highlights the expanding role of surface chemistry and molecular functionalization in enhancing sensor performance.

Beyond individual sensing platforms, the contribution by Soriano et al. [Contribution 7] addresses multidomain molecular sensor devices, systems, and algorithms for improved physiological monitoring. Their work emphasizes the importance of integrating sensors with intelligent data processing and algorithmic analysis to create comprehensive physiological monitoring systems. Such multidomain approaches represent an important direction for future wearable, implantable, and real-time healthcare technologies.

Collectively, the contributions in this issue reflect several important trends in modern sensor science. First, nanostructured materials continue to play a decisive role in improving analytical sensitivity, selectivity, and miniaturization. Second, interdisciplinary integration—combining materials science, electrochemistry, optics, computational simulation, electronics, and algorithm development—is increasingly necessary for addressing complex analytical challenges. Third, the convergence of environmental monitoring, biomedical diagnostics, and physiological sensing underscores the broad societal impact of advanced sensor technologies.

The editors would like to express sincere appreciation to all authors for their valuable contributions and to the reviewers for their careful evaluations and constructive comments, which ensured the high scientific quality of the published works. We also acknowledge the editorial and production teams of *Micromachines* for their continuous support throughout the publication process.

We hope that the studies presented in this collection will inspire further research and collaboration in the rapidly evolving field of nanostructured and molecular sensing technologies. The advances reported here demonstrate the substantial potential of innovative sensor systems to address pressing environmental, biomedical, and technological challenges in the years ahead.

It was really an honor for me to be a guest editor in a Special Issue where all these distinguished scientists have contributed. I wish to thank them all for their excellent cooperation.
